# Experimental Study of the Oriented Immobilization of Antibodies on Photonic Sensing Structures by Using Protein A as an Intermediate Layer

**DOI:** 10.3390/s18041012

**Published:** 2018-03-28

**Authors:** Raffaele Caroselli, Javier García Castelló, Jorge Escorihuela, María José Bañuls, Ángel Maquieira, Jaime García-Rupérez

**Affiliations:** 1 Nanophotonics Technology Center, Universitat Politècnica de València, Camino de Vera s/n, 46022 Valencia, Spain; rcaroselli@ntc.upv.es (R.C.); jagarcas@gmail.com (J.G.C.); 2 Departamento de Química, Instituto Universitario de Investigación de Reconocimiento Molecular y Desarrollo Tecnológico (IDM), Universitat Politècnica de València, Universitat de València, 46022 Valencia, Spain; joresfu1@upvnet.upv.es (J.E.); mbpolo@upvnet.upv.es (M.J.B.); amaquieira@qim.upv.es (Á.M.)

**Keywords:** Integrated photonics, ring resonator, sensor, biosensing

## Abstract

A proper antibody immobilization on a biosensor is a crucial step in order to obtain a high sensitivity to be able to detect low target analyte concentrations. In this paper, we present an experimental study of the immobilization process of antibodies as bioreceptors on a photonic ring resonator sensor. A protein A intermediate layer was created on the sensor surface in order to obtain an oriented immobilization of the antibodies, which enhances the interaction with the target antigens to be detected. The anti-bovine serum albumin (antiBSA)-bovine serum albumin (BSA) pair was used as a model for our study. An opto-fluidic setup was developed in order to flow the different reagents and, simultaneously, to monitor in real-time the spectral response of the photonic sensing structure. The antiBSA immobilization and the BSA detection, their repeatability, and specificity were studied in different conditions of the sensor surface. Finally, an experimental limit of detection for BSA recognition of only 1 ng/mL was obtained.

## 1. Introduction

In the last decade, integrated photonic sensors have been a significant object of research and have attracted interest in the chemical, biological, and medical fields [1,2,3,4,5,6]. This high interest is mainly due to their extremely high sensitivity and reduced size, which permits the integration of several sensing structures on a single chip for an ultra-sensitive multiplexed detection [1,7,8,9,10,11,12,13,14]. Furthermore, photonic sensors allow the development of label-free assays where the use of radioactive, colorimetric, or fluorescent labels, which introduce complexity and loss of activity to biological molecules, is avoided [15,16,17,18,19]. Therefore, advanced label-free biosensors can provide selective, sensitive, fast, and direct detection of a certain target analyte at extremely low concentrations.

A fundamental step to set up a label-free biosensor is the bio-functionalization of the sensor surface with a layer of bioreceptors able to “identify” the target analytes [20]. In fact, in this way it is possible to confer to the photonic structure the desired specificity for a particular target analyte. Therefore, determining the optimal surface modification, which means finding a suitable coating for the immobilization of the receptors onto the surface, is crucial for the development of a sensitive and selective biosensor and influences the final performance of the device [21,22].

In this work, we report the study of the process of the antibodies’ immobilization on a photonic sensing structure. The bio-functionalization approach is based on the use of an intermediate layer of protein A able to properly orient the antibody receptors, which is translated into a better sensitivity of the photonic structure. We focused in a well-known antibody-antigen system typically used as model in experimental developments: anti bovine serum albumin (antiBSA)-bovine serum albumin (BSA). To achieve this aim, a ring resonator-based photonic sensing structure was used. An opto-fluidic setup was developed to flow the reagents over the ring resonator surface and, simultaneously, to monitor the spectral evolution in real-time. Several immobilization and experimental protocols were studied in order to determine, first, how the orientation of the antibody influences the BSA recognition capability and, then, the repeatability and specificity of the approaches. Finally, a limit of detection of 1 ng/mL BSA was experimentally determined.

## 2. Materials and Methods

### 2.1. Usefulness of Protein A for Antibody Immobilization

A ring resonator is characterized by the capability to measure variations of the refractive index and, consequently, to quantify this variation by converting it into a variation of the optical signal. However, it is not characterized by the attribute to specifically recognize the presence of a certain biological molecule. In order to confer this specificity to the photonic sensor, the surface of the sensor must be coated with a biological layer (or biolayer). The biolayer allows covering the sensing structure with appropriate bioreceptors to recognize the target analytes and repel the non-specific binding of biomolecules of the analyzed sample [23]. Among the several types of bioreceptors employed for the biofunctionalization of ring resonator sensors, antibodies are mostly used, since they provide high specificity and affinity [17,24]. One of the most common techniques employed to immobilize antibodies on the sensor surface is the direct physical adsorption, due to its simplicity and rapidity. However, a problem can introduce a loss of sensitivity of the sensor: the random orientation of the bioreceptors when they are directly immobilized on the sensing structure. This problem can provoke that the binding between the bioreceptors and the target analytes does not take place or takes place in a wrong way, lowering the binding capacity in comparison to that in solution. Nowadays, in order to overcome this problem and obtain a highly-sensitive structure, other immobilization techniques to create a robust biolayer over the sensor surface have been developed including monolayers and covalent binding [17].

**Figure 1:**
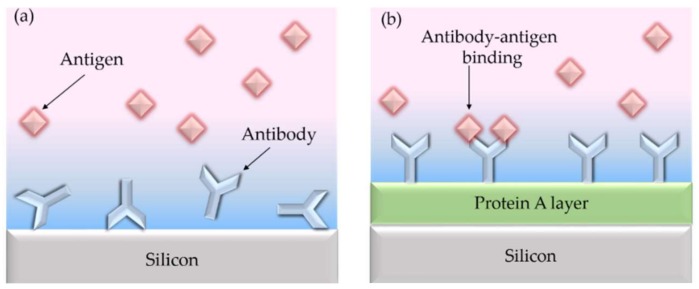
(**a**) Illustration of randomly-oriented antibody receptors on the surface of the sensor when they are directly immobilized using physical adsorption. The antibodies will not properly expose their Fab binding sites towards the sample solution with the target analytes; (**b**) Illustration of properly-oriented antibody receptors on the surface of the sensor where an intermediate layer of protein A was used. Antibodies will bind to the protein A layer through their Fc site, thus leaving their Fab sites oriented towards the sample solution with the target analytes.

In this work, the bio-functionalization approach to be used is based on the immobilization of the antibodies on the sensor surface through an additional intermediate layer of protein A, so that the antibodies can be anchored to the sensor surface by binding with this intermediate layer, as it is schematically depicted in Figure 1. Protein A is a 64 kDa microbial surface protein derived from the cell wall of *Staphylococcus aureus* [25]. It is an Fc-receptor biomolecule since it contains four binding sites specific to the Fc region of antibodies. Therefore, it specifically binds with the Fc region of immunoglobulin from many mammals, while leaving the Fab regions available for antigen binding. In this way, this bio-functionalization approach will allow that the anchored antibodies will be oriented in such way that the binding sites are exposed towards the sample solution containing the target analyte to be detected, thus maximizing the interaction with them [26]. Furthermore, since protein A provides four binding sites, it does not need a specific orientation to allow the oriented antibody immobilization. For this reason, the creation of the protein A layer can be achieved by physical adsorption, allowing to perform all the immobilization process in flow and, thus, monitoring it in real-time. This makes such a technique very simple and quick for the bio-functionalization of photonic structures. Additionally, once the antibodies are bound to the protein A, it is possible to break this binding by using a certain reagent in order to bio-functionalize again with a new batch of antibodies of the same kind and repeat the experiment or to attach another type of antibodies and carry out a different experiment [27].

### 2.2. Experimental Procedure

The sensing experiments carried out to validate the effective usefulness of the intermediate layer of protein A for the bio-functionalization of photonic sensing structures were focused on the detection of BSA as the target analyte. This antigen is part of the family of the albumins, which are globular proteins that are soluble in water and moderately soluble in concentrated salt solutions. BSA is characterized by a molecular weight of 66.5 kDa.

**Figure 2:**
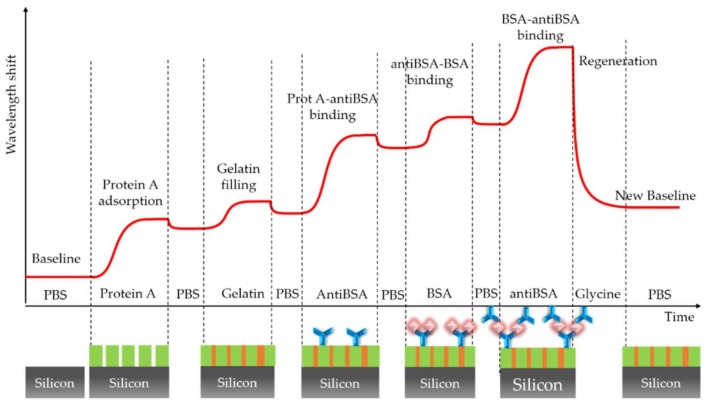
Schematic representation of the wavelength shift evolution of the whole experiment.

All the experiments were carried out exclusively in the flow. The standard experimental sensing procedure, whose wavelength shift evolution is shown in Figure 2, began with the flow of protein A with a concentration of 10 µg/mL over the ring resonator sensor in order to create, by physical adsorption, the intermediate protein A layer for a better immobilization of the bioreceptors, as previously explained. PBS 0.1× (phosphate-buffered saline, 0.8 mM sodium phosphate dibasic, 0.2 mM sodium phosphate monobasic, 13.7 mM sodium chloride, 0.3 mM potassium chloride, pH 7.5) was initially used as the buffer for the protein A (as well as for all the reagents to be flowed over the sensor surface). This buffer provides a pH very close to that for the optimal binding of the antibody to the protein A (pH = 8.2, with high affinity (K_a_ = 10^8^ M^−1^)). After creating the protein A intermediate layer, it is possible that not all of the sensor surface is completely covered with the protein, meaning that the bioreceptors can be adsorbed at those non-covered areas and remain there with a random orientation. In order to block the sensor surface, cold water fish skin gelatin (CWFS Gelatin) at a concentration of 100 µg/mL in PBS 0.1× was flowed [28]. This protein has low dimensions, it is extremely soluble in water, and is widely employed in immunoassays to block the surface against non-specific adsorption. Furthermore, the use of the gelatin allows us to test the proper creation of the protein A layer. If such a layer is consistent and well distributed, when the gelatin is flowing, it will not fill the layer and no wavelength shift will be observed. After this point, the buffer for the experiments was changed from pure PBS to PBS with gelatin at a concentration of 10 µg/mL, in order to maintain the blocking of the surface. Then, antiBSA with a concentration of 30 µg/mL was flowed. AntiBSA is the specific antibody for BSA and it is characterized by a molecular weight of 160 kDa, more than double that of BSA. Finally, in order to regenerate the sensor, at the end of every experiment, we flowed glycine, pH 2.5, at a concentration of 10 mM in PBS in order to break the interaction between protein A and antiBSA [29]. Glycine buffer solution, 100 mM, pH 2–2.5 is a solution commonly used for eluting antibodies in various antibody affinity purification procedures, such as protein A-agarose and protein G-agarose [30]. In this way, it was possible to restart the experiment with the chip functionalized with the protein A layer where we will bind again the antiBSA bioreceptors in order to carry out a new detection of BSA.

In order to confirm that the BSA was bound to the antiBSA, a sandwich assay was performed after the BSA detection cycle. This consisted of a second flow of antiBSA in order to observe an additional wavelength shift that confirms the presence of the BSA on the sensor surface.

In order to test the specificity of the assay, after protein A and antiBSA immobilization, ovalbumin (OVA) with a concentration of 100 ng/mL was flowed over the sensor instead of BSA as the target analyte. The ovalbumin is not the specific analyte of antiBSA and, thus, they do not recognize each other. In this way, no detection should be observed.

### 2.3. Photonic Structure

**Figure 3:**
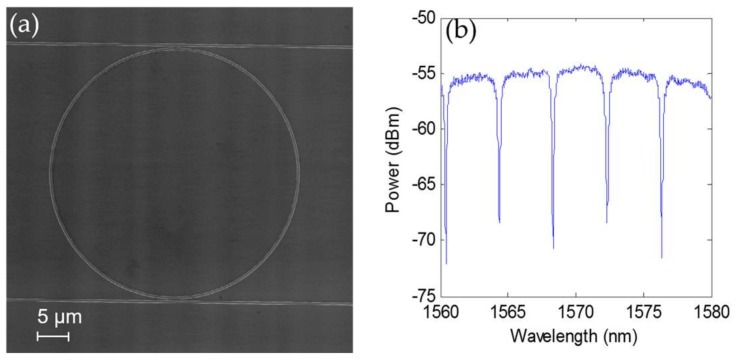
(**a**) SEM image of the ring resonator used for the experiments; (**b**) Transmission spectrum of the through port of the ring resonator.

The photonic structure used for our experiments is a SOI (silicon-on-insulator) ring resonator in the add-drop configuration, as shown in Figure 3, which was fabricated in CEA-LETI in the frame of the cost-shared European nanophotonic fabrication platform ePIXfab. These ring resonators are fabricated employing SOI wafers with a silicon thickness of 220 nm on top of a buried silicon oxide layer of 2 µm. The basic parameters of the sensing structures are: access waveguide width, 450 nm; ring radius, 20 µm; input coupling gap, 170 nm; and output coupling gap, 175 nm. The light coupling to the chip is carried out through shallow-etch access grating couplers.

### 2.4. Opto-Fluidic Setup

**Figure 4:**
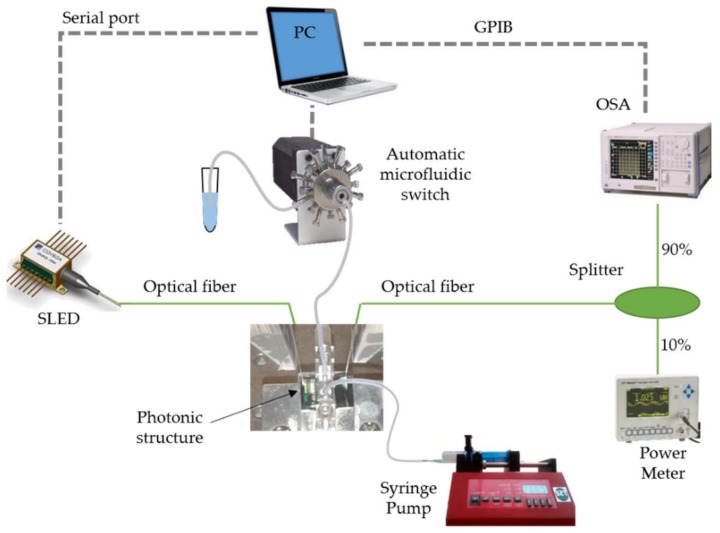
Scheme of the opto-fluidic setup used to carry out the sensing experiments.

A detailed characterization of the time evolution of a chemical reaction is key in order to understand whether a certain detection has occurred or not. To this aim, a real-time monitoring of the sensing structure response, while flowing the reagents on the sensor surface, is needed. To this aim, we developed an opto-fluidic experimental setup, which was able to carry out the sensing measurements in real-time. The setup was composed of fluidic and optical parts operating at the same time, in order to flow the reagents and, simultaneously, monitor the spectrum evolution. The whole experimental opto-fluidic setup is shown in Figure 4.

Regarding the optical part, a superluminescent diode (SLD) (SLED 8164 Covega, Thorlabs GmbH, Dachau, Germany), a broadband source with an optical bandwidth of 50 nm centered in 1550 nm, was used as the light source. Light from the SLD source was coupled to the photonic chip via a grating coupler using a cleaved optical fiber with an angle of 8°. A second cleaved optical fiber was used to collect the light from the output grating coupler of the photonic chip. The collected light was divided using a fiber 10–90 directional coupler: the 10% port was connected to a power meter (1931-C, Newport Corporation, Irvine, CA, USA) in order to monitor the output power during the fiber alignment process to obtain the best fiber placement, and the 90% port was connected to an optical spectrum analyzer (OSA) (AQ6317C, Ando Electric Co. Ltd., Tokio, Japan) in order to acquire the transmission spectrum of the photonic sensing structure. Data from the OSA were collected by means of GPIB acquisition data instrumentation and a LabVIEW (National Instruments Corporation, Austin, TX, USA) customized program was developed in order to continuously scan the spectrum of the biosensing device.

Regarding the fluidic part, we used a basic microfluidic delivery system based on polydimethylsiloxane (PDMS) in order to flow all the reagents over the sensing structure. The PDMS microfluidic channel had a length of 6 mm, a width of 400 µm and a height of 50 µm. The reagents were flowed with the help of a syringe pump (540060, TSE Systems, Bad Homburg, Germany). The pump was set to withdraw mode in order to aspirate the liquids from a vial located at the end of the microfluidic system, thus making the liquid to flow through the PDMS microchannel. Working in withdraw mode allows changing the liquid to be flowed by simply changing the vial at the end of the system, something that is not possible when working with the pump in injection mode. The pump was set to a constant flow rate of 10 µL/min during the whole experiment. Finally, the change between the several different liquids to be flowed during the experiments was made by using a multiposition automated switch (C25Z, VICI Valco Instruments Co. Inc., Houston, TX, USA) that permitted changing from 10 vessels to a common port using an electrical actuator.

## 3. Results

Several tests were performed by using the same photonic structure. In the first test, a bare ring resonator was bio-functionalized by directly flowing antiBSA on the surface without the use of the intermediate layer of protein A; these results will be used for comparison purposes in order to determine the performance enhancement produced by the use of the protein A intermediate layer. The second test made use of the protein A intermediate layer to immobilize the antiBSA probes over the photonic sensor. In the third test, the whole experimental procedure described in Section 2.2 was carried out twice, consecutively. In the fourth test, the specificity test was performed. Finally, in the last test, the limit of detection for BSA recognition was determined.

As explained in Section 2.2, we flowed the buffer after each step so that, after the dissociation process, it was possible to study the actual layer creation. For this reason, for each step, we considered, as the real wavelength shift, the residual shift after the dissociation process.

### 3.1. BSA Sensing without the Protein A Layer

**Figure 5:**
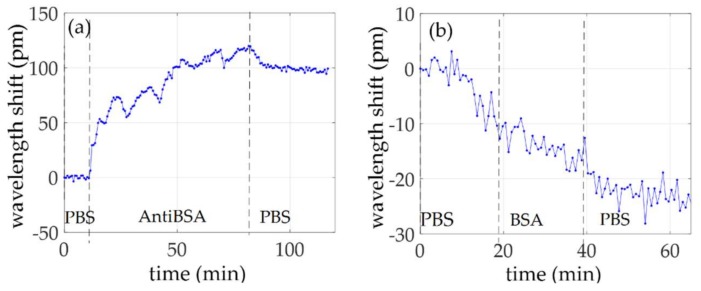
Wavelength shift time evolution of the experiment carried out without bio-functionalizing with protein A the ring resonator surface: (**a**) antiBSA adsorption; and (**b**) BSA 100 ng/mL flow.

AntiBSA was flowed directly over the bare ring resonator surface. Figure 5 shows the wavelength shift time evolution relative to the flow of (a) antiBSA and (b) BSA at a concentration of 100 ng/mL. AntiBSA was flowed for 70 min and BSA for 20 min. The antibody layer deposited on the surface caused a wavelength shift of around 100 pm and no BSA detection was observed. More in detail, during the evolution relative to antiBSA, a nonhomogeneous rise can be observed, which means a difficulty for the adsorption of the antiBSA until a plateau was reached. When the buffer started to flow again, a slight antiBSA desorption can be observed. When BSA was flowing, a negative drift was observed, meaning that antiBSA was still desorbing, until a plateau is reached.

### 3.2. BSA Sensing with a Protein A Intermediate Layer

**Figure 6:**
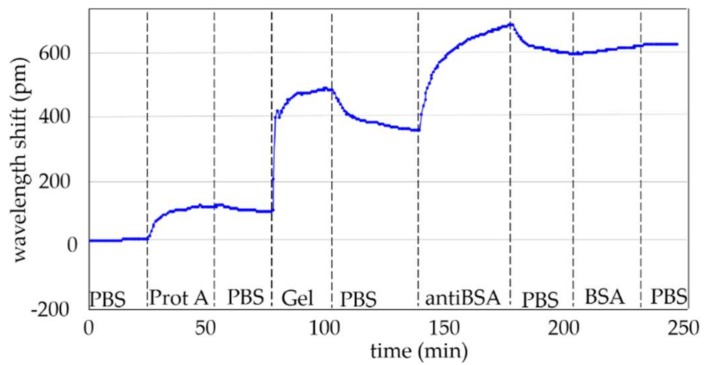
Wavelength shift time evolution of the whole experiment carried out by following the experimental procedure until BSA detection.

The second test was carried out by following the experimental procedure described in Section 2.2 until the BSA detection step. The aim of this experiment is to test the protein A layer creation and its effect over the immobilization of the antibodies. In Table 1, the flow times of the reagents are reported. In Figure 6, the wavelength shift time evolution is shown, where the creation of each layer (i.e., protein A, gelatin blocking, antiBSA and BSA) can be clearly appreciated. In this measurement, after the immobilization of the protein A, a wavelength shift of 110 pm was observed whereas, for the gelatin blocking step, the shift was around 270 pm. This means that the sensor surface was not fully covered by the protein A and the intermediate layer had defects. After flowing gelatin (Gel in Figure 6), during the PBS washing, a strong and constant negative drift can be appreciated. The drift represents the desorption of the gelatin. Next, a shift of about 220 pm was measured after the antiBSA immobilization. At the end, we flowed the target BSA with a concentration of 100 ng/mL in order to carry out the detection. The wavelength shift due to BSA was around 40 pm.

It is worth noting again the poor coverage of the protein A layer over the sensor, which is clearly indicated by the significantly higher shift measured for the gelatin blocking step. The reason for this poor coverage is the repulsion between the protein A, which is negatively charged for these pH conditions, and the thin layer of native silicon oxide present at the sensor surface, which is also negatively charged. As a consequence, a reduction of the surface adsorption degree of the protein A over the surface is produced.

### 3.3. BSA Sensing with a Protein A Intermediate Layer under Several Regeneration Cycles

Using the regeneration strategy described in Section 2.2, several experiments were consecutively carried out monitoring in real-time the evolution of the ring resonator response. In this way, once the intermediate protein A layer was created, it was possible to perform all the tests by using the same photonic structure covered by the same protein A layer. As a matter of fact, the glycine breaks the binding between protein A and antiBSA and the protein A layer is supposed to be completely preserved.

**Figure 7:**
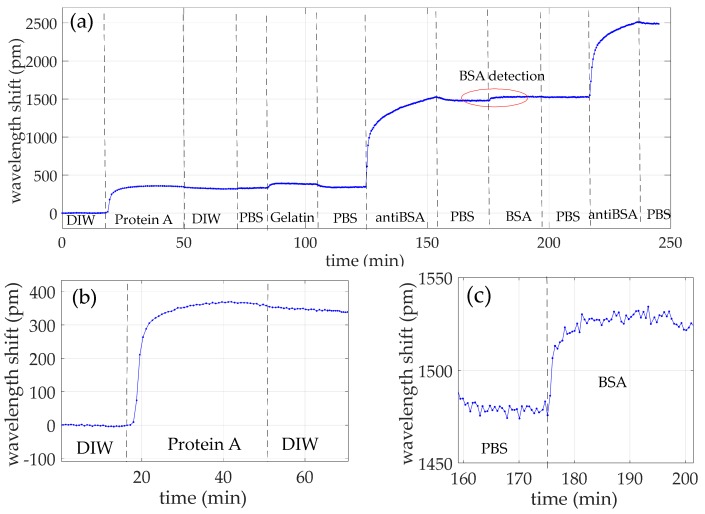
Wavelength shift time evolution of (**a**) the whole experiment carried out by following our experimental procedure; (**b**) the protein A adsorption; and (**c**) the BSA 10 ng/mL detection.

The first experiment was carried out by following the whole experimental procedure described in Section 2.2. However, DIW was used now as buffer for the protein A instead of PBS, in order to avoid the adsorption limitation previously described and to enhance the coverage of the sensor surface. By using DIW as a buffer, whose pH is 5–5.5, protein A is electrically neutral and its adsorption on the sensor surface can occur without charge-related limitations. Then, before running the gelatin blocking step, the buffer is changed from DIW to PBS. In Table 2, the flow times of the reagents are reported. In Figure 7a, the wavelength shift evolution of the experiment is shown. As shown in Figure 7b, the immobilization of protein A produced a wavelength shift of about 340 pm. Then, the gelatin blocking step caused a shift of only 5 pm, thus indicating that the protein A layer was properly created in the previous step. Next, a shift of around 1100 pm was measured for the antiBSA immobilization, five times higher than for the antiBSA immobilization step from the previous experiment. This result was due to the presence of the closely-packed protein A layer and confirms its effectiveness. At the end, the target BSA was flowed with a concentration of 10 ng/mL. A wavelength shift of around 50 pm was measured due to the recognition of the BSA, as shown in Figure 7c. In order to confirm such detection, the sandwich format was employed by flowing a second cycle of antiBSA. A wavelength shift around 950 pm was then measured, almost the same value as the first antiBSA flow, which indicated that almost all the BSA bound to the first antiBSA layer (capture antibody) was recognized by the second antiBSA flow (detection antibody).

**Figure 8:**
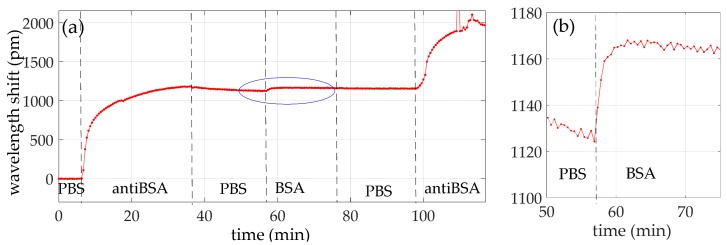
Wavelength shift time evolution of (**a**) the whole second experiment and (**b**) the BSA 10 ng/mL detection.

After the glycine regeneration process, a second biosensing experiment was carried out starting directly with the immobilization of the recognition antiBSA antibody. In Table 3, the flow times of the reagents are reported In Figure 8a, the wavelength shift evolution is shown. A wavelength shift due to the creation of the antiBSA layer of 1120 pm was observed. Then, BSA of 10 ng/mL detection produced a shift of about 45 pm, as shown in Figure 8b. Finally, the sandwich format caused a wavelength shift of around 800 pm. Such results are almost the same of those obtained in the previous experiment.

### 3.4. Specificity Test

**Figure 9:**
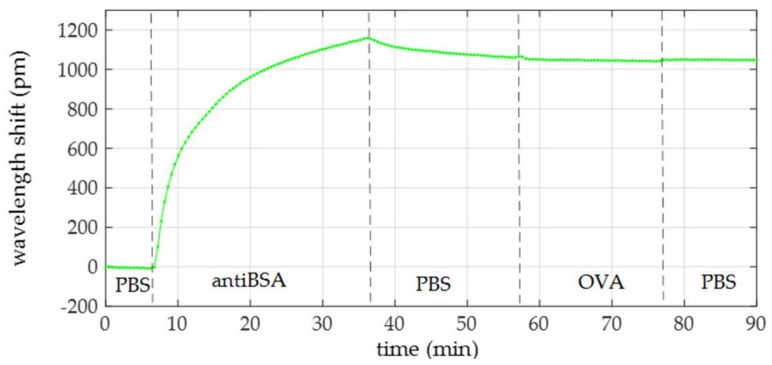
Wavelength shift time evolution of the OVA specificity test.

In order to demonstrate the specificity of this approach, we repeated the experiment once more, but OVA of 100 ng/mL was flowed as the target analyte instead of BSA. In Table 4, the flow times of the reagents are reported. Figure 9 shows the wavelength shift evolution of this specificity test. AntiBSA immobilization caused a shift of around 1070 pm, whereas no shift was observed when OVA was flowed.

### 3.5. Experimental Limit of Detection of BSA

**Figure 10:**
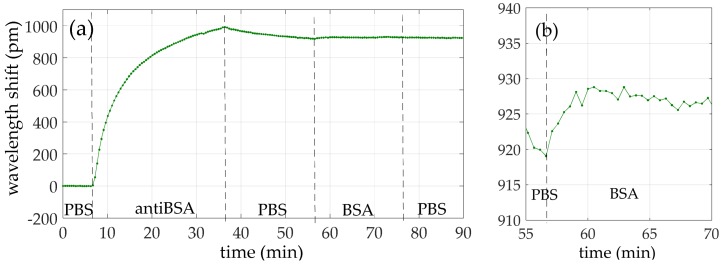
Wavelength shift time evolution of (**a**) the whole fifth experiment and (**b**) BSA 1 ng/mL detection.

Once the BSA detection was confirmed and the specificity of this approach was demonstrated with the previous experiments, additional experiments were carried out in order to determine the limit of detection of our sensor. In Table 5, the flow times of the reagents are reported. The lowest BSA concentration that we were able to detect was 1 ng/mL, which produced a wavelength shift of around 7 pm, as shown in Figure 10.

### 3.6. Sensitivity for AntiBSA and AntiBSA Detection Limit

In the experiments carried out using the same protein A layer, the wavelength shift caused by the antiBSA immobilization was determined. From that value, the sensitivity of the sensor and its minimum detectable total mass have been calculated.

The surface density of a closely-packed monolayer of antiBSA ρ_antiBSA_ is given by:

(1) $$\rho_{\mathrm{antiBSA}}=\frac{{\mathrm{MM}}_{\mathrm{antiBSA}}/N_{A}}{{\left(d_{\mathrm{antiBSA}}\right)}^{2}},$$

where MM_antiBSA_ is the molecular mass of antiBSA, N_A_ is the Avogadro’s number, and d_antiBSA_ is the average length of a molecule of antiBSA. Thus, taking into account that:

(2) $$\{\begin{array}{c} {\mathrm{MM}}_{\mathrm{antiBSA}}=160\;\mathrm{kDa}\\ N_{A}=6.02\cdot 10^{23}\\ d_{\mathrm{antiBSA}}=15\;\mathrm{nm} \end{array},$$

the antiBSA monolayer density will be ρ_antiBSA_ = 1.18 ng/mm^2^.

The sensitivity for antiBSA S_antiBSA_ is given by:

(3) $$S_{\mathrm{antiBSA}}=\frac{{\Delta \lambda }_{\mathrm{antiBSA}}}{\rho_{\mathrm{antiBSA}}},$$

where Δλ_antiBSA_ is the wavelength shift at antiBSA saturation. Since Δλ_antiBSA_ = 1120 pm, the sensitivity for antiBSA was S_antiBSA_ = 930 pm/ng/mm^2^.

The detection limit of the antiBSA surface mass density DL_ρ-antiBSA_ is given by:

(4) $${\mathrm{DL}}_{\rho -\mathrm{antiBSA}}=\frac{\sigma }{S_{\mathrm{antiBSA}}},$$

where σ is the noise observed in the experiments. From the sensing curves obtained in the experiments, we have determined a noise value of σ_PBS_ = 1 pm (during the flow of PBS before the antiBSA immobilization), leading to an antiBSA surface mass density detection limit of DL_ρ-antiBSA_ = 1.07 pg/mm^2^.

Furthermore, considering the surface area of the ring resonator A_RR_ = π · (R^2^ − r^2^) = 57.15 µm^2^, also the detection limit of the antiBSA total mass DL_antiBSA_ was calculated as:

(5) $${\mathrm{DL}}_{\mathrm{antiBSA}}{=\mathrm{DL}}_{\rho -\mathrm{antiBSA}}\times A_{\mathrm{RR}}=60\;\mathrm{ag}.$$

The calculated values are reported in Table 6.

## 4. Discussion

**Figure 11:**
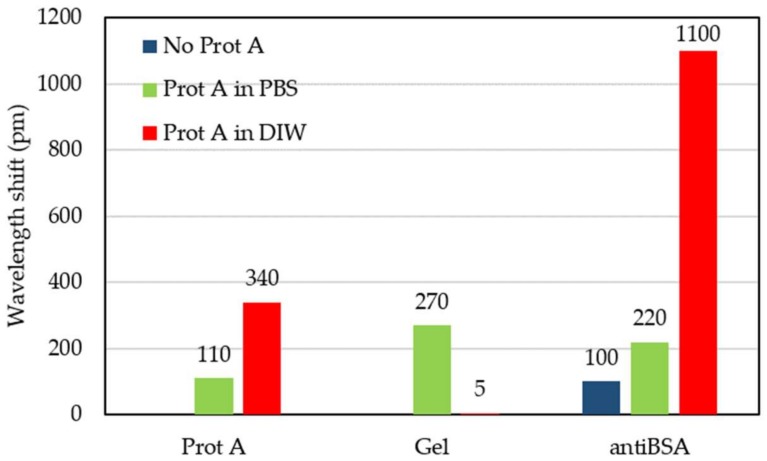
Wavelength shift for the different functionalization steps for the three immobilization protocols used in this work.

The experimental results confirm that the oriented immobilization of antiBSA took place by covering the ring resonator surface with the protein A intermediate layer. More in detail, a wavelength shift of around 340 pm was appreciated when flowing the protein A in DIW, more than three times higher than for the case when protein A was flowed in PBS, which allows having a consistent layer of protein A. Furthermore, such a protein A layer was closely packed, since a shift of only 5 pm was observed when gelatin was flowed. The presence of a well-coated protein A layer allowed an optimal immobilization of antiBSA, as a wavelength shift even higher than 1100 pm was observed and almost no antiBSA desorption was observed. Such a shift was around five times higher than for the case when the antiBSA was immobilized on a non-optimal protein A layer, thus confirming the importance of having a good protein A intermediate layer. Furthermore, this antiBSA shift was observed in all the four experiments performed using the same protein A layer. This result indicates that the protein A layer guarantees repeatability and its effectiveness does not decrease over time. In Table 7, the wavelength shifts obtained for the flow of the different reagents for the three protocols used are summarized, which are also graphically represented in Figure 11. It is possible to observe the large improvement obtained by flowing protein A in DIW. The detection limit of the antiBSA surface mass density and of the antiBSA total mass were only 1.07 pg/mm^2^ and 60 ag, respectively.

**Figure 12:**
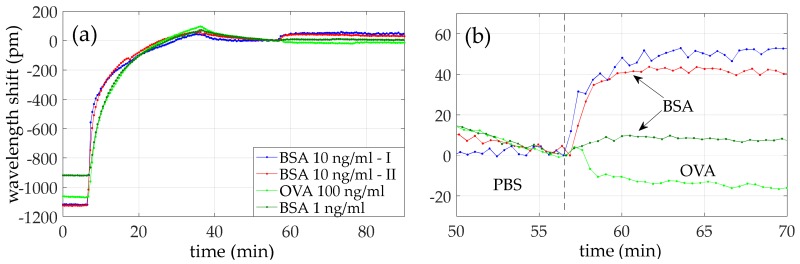
Comparison of the wavelength shift evolution of all the experiments: (**a**) antiBSA immobilization; and (**b**) target analyte detection.

**Figure 13:**
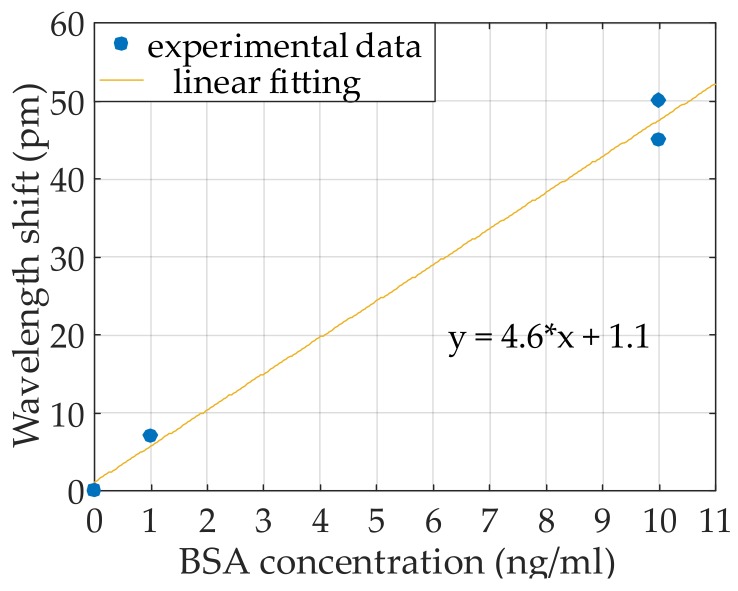
Wavelength shift values as a function of the BSA concentration.

The presence of a nonhomogeneous protein A layer led to the detection of BSA at 100 ng/mL with a wavelength shift of around 40 pm. On the contrary, the presence of a closely-packed protein A layer enhanced the interaction with the target antigens to be detected, as we were able to detect very low concentrations of BSA. As a matter of fact, BSA at 10 ng/mL was detected with a shift of around 45 pm, almost the same shift caused by BSA at 100 ng/mL in the previous experiment. The BSA recognition was confirmed by performing a sandwich assay by flowing an additional cycle of antiBSA, where a wavelength shift similar to that of the first antiBSA cycle was obtained, which demonstrates that the BSA was bound to almost all the Fab regions of the antiBSA on the surface. The specificity of this approach was also tested by flowing OVA as a target analyte. Figure 12 summarizes the sensing results obtained for the four experiments carried out using the same protein A layer. For a better comparison, a wavelength shift of 0 is considered for the moment when the target analytes begin to flow over the photonic sensor. As it can be clearly observed in Figure 12b, a shift is produced when BSA is flowed for all the concentrations, while no variation in the trend of the sensing signal is observed when OVA is flowed in the experiment. Figure 13 depicts the measured wavelength shift as a function of the target BSA concentration.

The reported results are comparable, or even better, to those previously reported in other works, with the advantage of an easier, faster, and cheaper antibody immobilization. Covalent binding is one of the most used immobilization techniques, due to the strong attachment of receptors to the photonic structure surface [24]. Very low detection limits have been achieved using this technique. For example, Xu et al. in [31] achieved a resolution of 40 ag of total mass. De Vos et al. in [32] achieved a limit of detection of 3.4 pm/mm^2^, with a theoretical minimum detectable total mass of 74 ag. In [33], Luchansky et al. reported a limit of detection of 1.5 pg/mm^2^. However, the protocol required to apply this immobilization technique is significantly more complex to that reported in this work. In addition to covalent binding, other techniques have been employed for the immobilization of oriented antibodies on ring resonators. For example, Taniguchi et al. used a silicon-binding protein based technique providing a sensitivity for biomaterial of 10 ng/mL [34]. Apart from the antibody immobilization, regarding the detection of BSA, Shan et al. reported a technique based on an organophosphonate surface coating and vinyl sulfone linker for the biofunctionalization of ring resonators for biomolecular sensing and detected BSA with a concentration of 250 ng/mL [35]. However, these techniques also require several steps, leading to large process times and more sample manipulation.

## 5. Conclusions

We have reported the experimental characterization of an antibody immobilization approach for ring resonator sensors based on the use of an intermediate layer of protein A to improve that immobilization. We tested the use of such a layer and confirmed its usefulness to obtain a well-coated surface of antibodies oriented towards the target sample to be analyzed, thus enabling the development of high sensitivity biosensors. The experimental validation was carried out by means of BSA detection experiments. By covering the sensor surface with a closely-packed intermediate protein A layer, antiBSA immobilization was considerably improved. Such immobilization improvement led to the detection of lower BSA concentrations, such as 1 ng/mL. Therefore, these results confirm the good performance in terms of sensitivity that can be obtained using a very simple, cheap, and easy-to-implement bio-functionalization approach. Furthermore, we proved the repeatability and the durability over time of this approach, since several experiments were carried out by using the same protein A layer and the same results were obtained. We also confirmed the specificity of the experimental procedure where we used gelatin as a blocking agent in order to avoid non-specific adsorption. Moreover, the creation of each layer was monitored in real-time thanks to the opto-fluidic setup developed to carry out all the experiments that we have reported.

Finally, this immobilization approach was very simple and fast, since the protein A layer was created over the ring resonator surface simply by physical immobilization. In fact, we were able to create such layer in a few minutes just by flowing protein A in DIW over the ring resonator surface, allowing the real-time monitoring of the layer creation.

## Figures and Tables

**Table 1:** Flow times of the reagents flowed in the experiment. The times are expressed in minutes.

PBS	Prot A	PBS	Gel	PBS	AntiBSA	PBS	BSA	PBS
20	35	20	25	40	40	20	20	10

**Table 2:** Flow times of the reagents flowed in the experiment. The times are expressed in minutes.

DIW	Prot A	DIW	PBS	Gel	PBS	AntiBSA	PBS	BSA	PBS	AntiBSA	PBS
15	35	20	10	25	20	30	20	20	20	20	10

**Table 3:** Flow times of the reagents flowed in the experiment. The times are expressed in minutes.

PBS	AntiBSA	PBS	BSA	PBS	AntiBSA
6	30	20	20	20	20

**Table 4:** Flow times of the reagents flowed in the experiment. The times are expressed in minutes.

PBS	AntiBSA	PBS	OVA	PBS
6	30	20	20	14

**Table 5:** Flow times of the reagents flowed in the experiment. The times are expressed in minutes.

PBS	AntiBSA	PBS	BSA	PBS
6	30	20	20	14

**Table 6:** Sensitivity parameters calculated from the antiBSA immobilization step.

Δλ_antibsa_ (pm)	S_antiBSA_ (pm/ng/mm^2^)	σ_PBS_ (pm)	DL_ρ-antiBSA_ (pm/mm^2^)	DL_antiBSA_ (ag)
1120	930	1	1.07	60

**Table 7:** Wavelength shift for the different functionalization steps for the three biofunctionalization protocols used in this work.

Protocol	Wavelength Shift (pm) Due to:		
	Protein A	Gelatin	AntiBSA
No protein A layer	Not flowed	Not flowed	100
Protein A in PBS	110	270	220
Protein A in DIW	340	5	1100

## References

[1] Huertas C.S., Fariña D., Lechuga L.M. (2016). Direct and Label-Free Quantification of Micro-RNA-181a at Attomolar Level in Complex Media Using a Nanophotonic Biosensor. ACS Sens..

[2] Estevez M.C., Alvarez M., Lechuga L.M. (2012). Integrated optical devices for lab-on-a-chip biosensing applications. Laser Photonics Rev..

[3] Luchansky M.S., Bailey R.C. (2012). High-Q optical sensors for chemical and biological analysis. Anal. Chem..

[4] Qavi A.J., Kindt J.T., Gleeson M.A., Bailey R.C. (2011). Anti-DNA: RNA antibodies and silicon photonic microring resonators: Increased sensitivity for multiplexed microRNA detection. Anal. Chem..

[5] Šípová H., Zhang S., Dudley A.M., Galas D., Wang K., Homola J. (2010). Surface plasmon resonance biosensor for rapid label-free detection of microribonucleic acid at subfemtomole level. Anal. Chem..

[6] Fan X., White I.M., Shopova S.I., Zhu H., Suter J.D., Sun Y. (2008). Sensitive optical biosensors for unlabeled targets: A review. Anal. Chim. Acta.

[7] García-Rupérez J., Toccafondo V., Bañuls M.J., Castelló J.G., Griol A., Peransi-Llopis S., Maquieira Á. (2010). Label-free antibody detection using band edge fringes in SOI planar photonic crystal waveguides in the slow-light regime. Opt. Express.

[8] Toccafondo V., García-Rupérez J., Bañuls M.J., Griol A., Castelló J.G., Peransi-Llopis S., Maquieira A. (2010). Single-strand DNA detection using a planar photonic-crystal-waveguide-based sensor. Opt. Lett..

[9] Passaro V.M.N., Troia B., La Notte M., Leonardis F (2013). De Photonic resonant microcavities for chemical and biochemical sensing. RSC Adv..

[10] Scullion M.G., Krauss T.F., Di Falco A. (2013). Slotted photonic crystal sensors. Sensors.

[11] Lee M.R., Fauchet P.M. (2007). Two-dimensional silicon photonic crystal based biosensing platform for protein detection. Opt. Express.

[12] Buswell S.C., Wright V.A., Buriak J.M., Van V., Evoy S. (2008). Specific detection of proteins using photonic crystal waveguides. Opt. Express.

[13] Castello J.G., Toccafondo V., Perez-Millan P., Losilla N.S., Cruz J.L., Andres M.V., Garcia-Ruperez J. (2011). Real-time and low-cost sensing technique based on photonic bandgap structures. Opt. Lett..

[14] Patko D., Cottier K., Hamori A., Horvath R. (2012). Single beam grating coupled interferometry: High resolution miniaturized label-free sensor for plate based parallel screening. Opt. Express.

[15] Zanchetta G., Lanfranco R., Giavazzi F., Bellini T., Buscaglia M. (2017). Emerging applications of label-free optical biosensors. Nanophotonics.

[16] Gavela A.F., García D.G., Ramirez J.C., Lechuga L.M. (2016). Last advances in silicon-based optical biosensors. Sensors.

[17] Hunt H.K., Armani A.M. (2010). Label-free biological and chemical sensors. Nanoscale.

[18] Vollmer F., Yang L., Fainman S. (2012). Label-free detection with high-Q microcavities: A review of biosensing mechanisms for integrated devices. Nanophotonics.

[19] Peter B., Farkas E., Forgacs E., Saftics A., Kovacs B., Kurunczi S., Szekacs I., Csampai A., Bosze S., Horvath R. (2017). Green tea polyphenol tailors cell adhesivity of RGD displaying surfaces: Multicomponent models monitored optically. Sci. Rep..

[20] Escorihuela J., Bañuls M.J., Castelló J.G., Toccafondo V., García-Rupérez J., Puchades R., Maquieira Á. (2012). Chemical silicon surface modification and bioreceptor attachment to develop competitive integrated photonic biosensors. Anal. Bioanal. Chem..

[21] Rusmini F., Zhong Z., Feijen J. (2007). Protein immobilization strategies for protein biochips. Biomacromolecules.

[22] Bañuls M.J., Puchades R., Maquieira Á. (2013). Chemical surface modifications for the development of silicon-based label-free integrated optical (IO) biosensors: A review. Anal. Chim. Acta.

[23] Jonkheijm P., Weinrich D., Schröder H., Niemeyer C.M., Waldmann H. (2008). Chemical strategies for generating protein biochips. Angew. Chem. Int. Ed..

[24] Zourob M., Lakhtakia A. (2010). Optical Guided-Wave Chemical and Biosensors II.

[25] Anderson G.P., Jacoby M.A., Ligler F.S., King K.D. (1997). Effectiveness of protein A for antibody immobilization for a fiber optic biosensor. Biosens. Bioelectron..

[26] Wang Z., Jin G. (2003). Feasibility of protein A for the oriented immobilization of immunoglobulin on silicon surface for a biosensor with imaging ellipsometry. J. Biochem. Biophys. Methods.

[27] Meyer V.K., Kober C., Niessner R., Seidel M. (2015). Regeneration of recombinant antigen microarrays for the automated monitoring of antibodies against zoonotic pathogens in swine sera. Sensors.

[28] Julián E., Cama M., Martínez P., Luquin M. (2001). An ELISA for five glycolipids from the cell wall of Mycobacterium tuberculosis: Tween 20 interference in the assay. J. Immunol. Methods.

[29] Lebogang L., Mattiasson B., Hedström M. (2014). Capacitive sensing of microcystin variants of Microcystis aeruginosa using a gold immunoelectrode modified with antibodies, gold nanoparticles and polytyramine. Microchim. Acta.

[30] Ngo T.N. (2013). Molecular Interactions in Bioseparations.

[31] Xu D.-X., Vachon M., Densmore A., Ma R., Delâge A., Janz S., Lapointe J., Li Y., Lopinski G., Zhang D., Liu Q.Y., Cheben P., Schmid J.H. (2010). Label-free biosensor array based on silicon-on-insulator ring resonators addressed using a WDM approach. Opt. Lett..

[32] De Vos K., Girones J., Claes T., De Koninck Y., Popelka S., Schacht E., Baets R., Bienstman P. (2009). Multiplexed antibody detection with an array of silicon-on-insulator microring resonators. IEEE Photonics J..

[33] Luchansky M.S., Washburn A.L., Martin T.A., Iqbal M., Gunn L.C., Bailey R.C. (2010). Characterization of the evanescent field profile and bound mass sensitivity of a label-free silicon photonic microring resonator biosensing platform. Biosens. Bioelectron..

[34] Taniguchi T., Hirowatari A., Ikeda T., Fukuyama M., Amemiya Y., Kuroda A., Yokoyama S. (2016). Detection of antibody-antigen reaction by silicon nitride slot-ring biosensors using protein G. Opt. Commun..

[35] Shang J., Cheng F., Dubey M., Kaplan J.M., Rawal M., Jiang X., Newburg D.S., Sullivan P.A., Andrade R.B., Ratner D.M. (2012). An organophosphonate strategy for functionalizing silicon photonic biosensors. Langmuir.

